# The association between teachers’ growth mindset and job satisfaction: the chain mediating role of teacher-student relationship and teacher self-efficacy

**DOI:** 10.3389/fpsyg.2026.1864396

**Published:** 2026-07-02

**Authors:** Zhengzhou Zhu, Yuxuan Bao, Ziying Zhang, Yipu Hao

**Affiliations:** 1 School of Education, Central China Normal University, Wuhan, China; 2 Wuhan Xinhe Street School, Wuhan, China; 3 Normal School, Hubei University, Wuhan, China

**Keywords:** growth mindset, TALIS, teacher job satisfaction, teacher self-efficacy, teacher-student relationship

## Abstract

**Introduction:**

In recent years, teacher turnover rates have risen significantly, posing a major challenge to efforts to improve educational quality. Teacher job satisfaction is a key factor influencing teacher turnover. This study applies Self-Determination Theory to explore the indirect associations of teacher-student relationship and self-efficacy in the relationship between teachers’ growth mindset and job satisfaction.

**Methods:**

Data were drawn from 1,201 teachers in Shanghai who participated in the OECD’s 2024 Teaching and Learning International Survey (TALIS). Validated scales assessed growth mindset, job satisfaction, teacher-student relationship quality, and self-efficacy. Structural equation modeling and bootstrap analyses were conducted to test direct and indirect effects.

**Results:**

Teachers’ growth mindset is significantly and positively correlated with job satisfaction, teacher-student relationship, and self-efficacy. Both teacher-student relationship and self-efficacy are positively correlated with teacher job satisfaction. Teacher-student relationship is positively correlated with teacher self-efficacy. Teacher-student relationship and self-efficacy not only mediate the relationship between teachers’ growth mindset and job satisfaction independently but also exert a chained mediating effect.

**Conclusion:**

These findings underscore that fostering a growth mindset is associated with higher teacher job satisfaction by first enriching teacher-student relationship and subsequently strengthening teachers’ sense of competence. Schools and policymakers should implement integrated interventions, such as cultivating growth mindsets, promoting positive teacher-student interactions, and bolstering self-efficacy, to create a virtuous cycle that sustains teachers job satisfaction.

## 1 Introduction

Teacher job satisfaction, a key indicator of teachers’ professional well-being and educational quality, has long attracted extensive attention from educational researchers (Sahito and Vaisanen, 2020). A large body of research indicates that teacher job satisfaction not only directly influences teachers’ instructional performance (Harrison et al., 2023), teaching confidence (Baluyos et al., 2019), and work enthusiasm (Burić and Moè, 2020), but is also closely related to teachers’ professional burnout and intention to leave (Kelly et al., 2019). When teachers feel dissatisfied with their work, not only is their own mental health compromised, but it may also trigger a negative chain reaction affecting students’ academic achievement and holistic development (Baluyos et al., 2019). Therefore, exploring the factors associated with teacher job satisfaction and the mechanisms by which they operate holds significant theoretical and practical value.

Previous research indicates that teacher job satisfaction is influenced by a combination of environmental, economic, social, and psychological factors (Lopes and Oliveira, 2020; Iwu et al., 2018). One important psychological factor is mindset, which refers to the belief that one’s abilities are either fixed or malleable (Dweck and Yeager, 2019). Individuals with a fixed mindset view ability as a fixed, innate trait that cannot be changed, whereas those with a growth mindset perceive ability as composed of continuously expanding knowledge and skills that can be altered through effort (Dweck and Yeager, 2019). Multiple studies have shown a positive correlation between a growth mindset and job satisfaction (Nalipay et al., 2019; Vettori et al., 2022), but their limitations have hindered a deeper understanding of this relationship. First, there is a significant gap in the literature on the relationship between a growth mindset and job satisfaction among Chinese teachers. Given the prevalence of low job satisfaction among Chinese teachers and its negative consequences (Liu et al., 2023), there is an urgent need to examine this association directly. Second, prior research has rarely examined the underlying psychological mechanisms by which a growth mindset enhances job satisfaction. Investigating these mechanisms may provide insights for developing potential interventions to improve teachers’ job satisfaction.

To address the limitations of existing research, this study adopts Self-Determination Theory as its explanatory framework. As a macro-level theory of human motivation and personality, Self-Determination Theory posits that the driving force behind individual behavior primarily stems from the fulfillment of three core psychological needs: competence, relatedness, and autonomy (Li et al., 2025). The fulfillment of these needs can stimulate intrinsic motivation, thereby enhancing behavioral engagement and emotional well-being. Specifically, the need for competence refers to an individual’s perception, during interactions with the social environment, that they possess the ability to complete tasks and can effectively control task outcomes; this perception fosters a sense of self-efficacy (Deci et al., 2017). The need for relatedness, on the other hand, refers to an individual’s need to establish connections with others, be cared for, and care for others, thereby forming meaningful social bonds (Li et al., 2025). Accordingly, this study examines a sample of teachers in Shanghai, China, to explore the relationship between a growth mindset and teacher job satisfaction. It focuses on investigating the underlying psychological mechanisms, specifically the mediating roles of teacher-student relationship (a manifestation of relational needs) and teacher self-efficacy (a manifestation of competence needs), thereby deepening our understanding of how a growth mindset relates to outcome variables and providing empirical evidence for understanding how teacher job satisfaction is associated with stimulating their fundamental psychological needs.

Despite the growing body of literature on the relationship between growth mindset and teacher job satisfaction, this study makes several distinctive contributions. First, most existing evidence has been derived from Western cultural contexts, leaving the applicability of these findings in non-Western educational settings—particularly in Shanghai, a high-performing yet high-pressure education system—largely untested. By empirically examining the association between growth mindset and job satisfaction using data from Shanghai teachers, this study addresses a critical gap in the Chinese context. Second, while previous research has largely treated the teacher-student relationship and self-efficacy as independent mediators, the potential chained-mediated mechanism between the two has been largely overlooked. This study proposes and tests a chained mediation model, thereby not only enriching the existing framework of underlying mechanisms but also offering valuable practical insights. Third, unlike prior studies that often rely on small or convenience samples, this study leverages large-scale, high-quality international survey data (TALIS 2024), thereby enhancing the robustness and generalizability of the findings. Collectively, these contributions advance our understanding of how a growth mindset relates to teacher job satisfaction and guide policy practices across diverse educational contexts.

## 2 Literature review and research hypotheses

### 2.1 Growth mindset and teacher job satisfaction

Implicit theories (or mindsets) refer to people’s beliefs about whether personal traits (such as intelligence or ability) are fixed or malleable, corresponding to growth and fixed mindsets, respectively (Dweck et al., 1995). Individuals with a fixed mindset believe that intelligence and ability are stable, unchangeable traits; consequently, they feel there is no need to strive to develop them. Conversely, individuals with a growth mindset believe that intelligence and ability are traits that can be enhanced or improved through sustained effort, appropriate strategies, and guidance from others (Dweck, 2006). Those with a growth mindset welcome challenges, view difficulties as learning opportunities (Tseng et al., 2020), and are adept at devising effective strategies to overcome obstacles (Dweck and Yeager, 2019; Hochanadel and Finamore, 2015).

According to Locke (1969), job satisfaction is a positive emotional state resulting from a favorable evaluation of one’s work or work experience. Job satisfaction is a function of the relationship between an individual’s expectations of work and the value actually provided by the job, reflecting the outcome of the individual’s interaction with the work environment (Locke, 1969). In the TALIS study, teacher job satisfaction is specifically defined as the sense of accomplishment and fulfillment teachers derive from their work (Zakariya et al., 2020). This construct is multidimensional and typically comprises two key dimensions: first, satisfaction with the teaching profession itself, that is, an individual’s evaluation of the decision to become a teacher; and second, satisfaction with the current work environment, which involves evaluations of the school’s overall atmosphere, resources, and management. Teacher job satisfaction is crucial to the education system; it not only influences teacher self-efficacy, self-esteem, well-being, and professional commitment (Sahito and Vaisanen, 2020; Sumanasena and Mohamed, 2022) but is also closely related to teacher turnover rates, retention intentions, and teaching engagement (Madigan and Kim, 2021).

Existing research indicates that a growth mindset is a key predictor of teacher job satisfaction (Ronkainen et al., 2019). First, according to Implicit Theories, teachers with a growth mindset are more likely to adopt positive coping strategies when encountering setbacks or difficulties, thereby effectively overcoming negative emotions at work and exhibiting positive emotions and behaviors, which directly contribute to higher job satisfaction (Kapasi and Pei, 2022; Tang et al., 2022). Second, a growth mindset encourages teachers to adopt mastery-oriented strategies, which increase the likelihood of achieving career goals and enhance work efficacy, thereby leading to higher job satisfaction (Janssen and Van Yperen, 2004). Empirical research indicates a positive correlation between growth mindset and teacher job satisfaction (Nalipay et al., 2019). Based on the above analysis, the following hypothesis is proposed:

*H1*: Teachers’ growth mindset is significantly and positively associated with teacher job satisfaction.

### 2.2 Growth mindset, teacher-student relationship, and teacher job satisfaction

The teacher-student relationship refers to the quality of the psychological bond formed between teachers and students through daily educational interactions, as experienced by teachers, encompassing emotional connections and cognitive evaluations (Sabol and Pianta, 2012; Ye and Wang, 2024). This psychological bond constitutes a key interpersonal relationship for teachers and is crucial to fulfilling their basic psychological needs, particularly their need for relatedness (Deci et al., 2017). The teacher-student relationship is generally regarded as one of the primary reasons teachers remain committed to the field of education (Spilt et al., 2011). Extensive empirical research indicates that when teachers experience warm, supportive student-teacher relationships, their job satisfaction increases significantly (Heinla and Kuurme, 2024). Conversely, conflictual or negative relationships serve as major predictors of professional burnout and turnover intentions (Evans et al., 2019). From the perspective of self-determination theory, positive teacher-student relationship fulfill teachers’ relational needs for intimacy and mutual care. The fulfillment of these needs, in turn, stimulates intrinsic motivation and enhances professional well-being and the willingness to remain committed (Chatzistavridou et al., 2025). Conversely, if teacher-student relationship are strained and the need for relatedness is thwarted, teachers’ job satisfaction declines, and they may even become more likely to leave their positions (Evans et al., 2019). Therefore, the teacher-student relationship can be viewed as a key proximal variable explaining fluctuations in teachers’ job satisfaction.

Furthermore, teachers’ growth mindset, that is, the belief that their abilities and teaching skills can be developed through effort and learning, significantly influences the quality of the teacher-student relationship as they perceive and construct it (Liu et al., 2025). According to self-determination theory, teachers with a high growth mindset are more likely to proactively adjust their interaction styles, understand students’ emotions and inner worlds from the students’ perspective, and thereby form more supportive mental models. They are also more likely to seek feedback actively and reflect on their teaching practices, thereby establishing teacher-student bonds based on trust and respect (Liu et al., 2025). Conversely, teachers with a fixed mindset tend to attribute interaction difficulties to students or the environment. They are inclined to avoid constructive communication, leading to a teacher-student relationship characterized by alienation or conflict (Dweck et al., 1995). Therefore, based on the above literature, the following hypothesis is proposed:

*H2*: Teacher-student relationship is significantly and positively associated with teacher job satisfaction.

*H3*: Teachers’ growth mindset is significantly and positively associated with the teacher-student relationship.

*H4*: Teacher-student relationship mediates the relationship between teachers’ growth mindset and job satisfaction.

### 2.3 Growth mindset, teacher self-efficacy, and teacher job satisfaction

Self-efficacy refers to an individual’s belief in their ability to perform specific tasks (Bandura, 1978). For teachers, self-efficacy is typically defined as their assessment of their ability to effectively guide students and complete specific teaching tasks (Dellinger et al., 2008). Self-determination theory suggests that when an individual’s need for competence is met, intrinsic motivation and mental well-being are significantly enhanced (Deci et al., 2017). In educational contexts, teachers with high self-efficacy are more likely to view teaching challenges as controllable and surmountable, thereby experiencing a stronger sense of accomplishment and control over their work. Existing empirical literature also indicates a significant positive correlation between teacher self-efficacy and job satisfaction (Liu et al., 2023). Teachers with high self-efficacy not only have greater confidence in their teaching abilities but are also more likely to derive positive emotions and a sense of professional value from their daily work, thereby enhancing their satisfaction with the work environment (Perera et al., 2018). Conversely, low self-efficacy tends to lead to professional burnout, emotional exhaustion, and negative evaluations of work, undermining job satisfaction (Zakariya and Wardat, 2024). Therefore, self-efficacy serves as a key mediating variable linking individual psychological resources to professional well-being (Chang and Sung, 2024).

According to Dweck’s implicit theories, a growth mindset also significantly enhances teacher self-efficacy (Dweck and Yeager, 2019). First, based on social cognitive theory, active mastery is an underlying source of self-efficacy (Bandura, 1986). Teachers with a growth mindset believe that human abilities are malleable; they actively strive toward their goals and cultivate a stronger sense of control. This active mastery serves as a prerequisite for self-efficacy (Dweck and Leggett, 1988). Second, teachers with a growth mindset are more open to feedback and professional learning, and they view technological pressures or classroom setbacks as opportunities for growth rather than signs of incompetence. This helps alleviate self-doubt and occupational anxiety, further reinforcing the belief that I am competent (Lin et al., 2022). Based on these relationships, it can be inferred that a growth mindset indirectly satisfies teachers’ intrinsic need for competence by enhancing their self-efficacy, thereby promoting increased job satisfaction. Based on this, the present study proposes the following hypothesis:

*H5*: Teacher self-efficacy is significantly and positively associated with teacher job satisfaction.

*H6*: Teachers’ growth mindset is significantly and positively associated with teacher self-efficacy.

*H7*: Teacher self-efficacy mediates the relationship between teachers’ growth mindset and job satisfaction.

### 2.4 Chain mediation of teacher-student relationship and teacher self-efficacy

Theoretically, the teacher-student relationship is regarded as a correlate of teacher self-efficacy. A positive teacher-student relationship provides teachers with warmth, support, and an emotional connection. In such interactions, students’ positive feedback, active participation, and emotional encouragement not only directly enhance teachers’ direct experience of teaching effectiveness but also shape teachers’ beliefs in their own abilities through verbal encouragement and emotional arousal (Hajovsky et al., 2020). At the same time, when teachers perceive a close relationship with students, they are more likely to obtain direct evidence of teaching success, thereby enhancing their sense of competence; and the fulfillment of this sense of competence is the core source of self-efficacy (Wang, 2025). Conversely, conflictual teacher-student relationship threaten a sense of belonging, trigger negative emotional arousal, and undermine teachers’ judgments of their own teaching abilities (Xu and Qi, 2019). In light of the above research, the following hypothesis is proposed:

*H8*: Teacher-student relationship is significantly and positively associated with teacher self-efficacy.

*H9*: Teacher-student relationship and teacher self-efficacy play a chain-mediating role between teachers’ growth mindset and job satisfaction.

**Figure 1:**
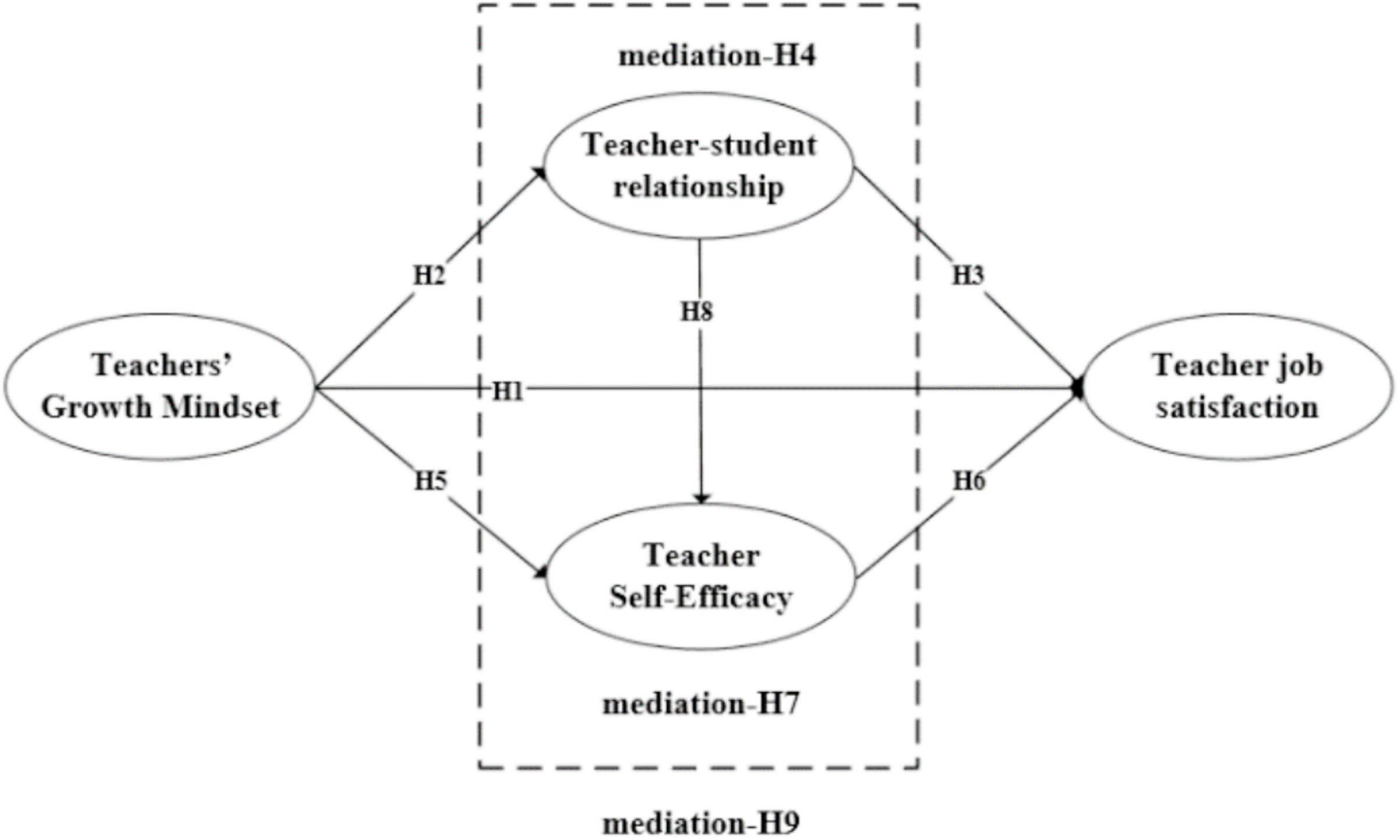
Research framework.

Based on the above hypothesis, this study constructs a research framework, as shown in Figure 1.

## 3 Methods

### 3.1 Data sources

The data used in this study were drawn from the Teaching and Learning International Survey (TALIS 2024) conducted by the Organization for Economic Co-operation and Development (OECD) in 2024. TALIS 2024 is a large-scale international comparative study of primary and secondary school teachers and principals, designed to systematically examine educators’ individual characteristics, the teacher-student relationship, and teaching practices. In Shanghai, China, the survey employed a stratified random sampling design: schools were first selected, and then teachers were randomly selected from the participating schools. The questionnaire was administered online, and teachers participated voluntarily based on informed consent. The questionnaire underwent a rigorous translation and back-translation process to ensure cross-cultural equivalence.

To ensure data quality, and in accordance with existing research (Qian and Yan, 2026), this study conducted systematic data cleaning. First, samples with missing values in key demographic variables (such as gender and years of teaching experience, with missing rates both less than 2%) were excluded. Second, given the skip logic embedded in the TALIS questionnaire (which omits certain subsequent scales based on responses to screening questions), only respondents who provided complete answers for the core indicators required for this study were retained. Third, outlier detection was applied to continuous variables to exclude extreme values falling outside a reasonable range. Following these procedures, the final analysis sample comprised 1,201 valid cases, meeting the requirements for multivariate statistical analysis. The sample included 923 female teachers (76.85%) and 278 male teachers (23.15%). Regarding years of teaching experience, 262 teachers (21.82%) had 5 years or less, 187 (15.57%) had 6–10 years, 316 (26.31%) had 11–20 years, and 436 (36.30%) had 20 years or more.

### 3.2 Measures

#### 3.2.1 Growth mindset

The Growth Mindset measurement is derived from the Growth Mindset Scale developed by Dweck et al. (1995). The Growth Mindset Scale in TALIS consists of three items. A sample item is: “Everyone has a fixed level of intelligence, and there is little anyone can do to really change it” (reversely coded). Each item is scored using a four-point Likert scale; higher scores indicate a stronger growth mindset. The Cronbach’s alpha coefficient for the Growth Mindset Scale is 0.863. Confirmatory factor analysis of the Growth Mindset Scale showed that standardized factor loadings ranged from 0.748 to 0.863, indicating that all items loaded strongly and consistently on their respective constructs.

#### 3.2.2 Job satisfaction

The teacher job satisfaction scale in TALIS 2024 comprises two dimensions: “Job satisfaction with work environment” and “Job satisfaction with profession,” consisting of a total of eight items. The work environment dimension includes four items, such as “I enjoy working at this school.” The job satisfaction dimension also includes four items, such as “The advantages of being a teacher clearly outweigh the disadvantages.” Items are measured using a four-point Likert scale (1 = Strongly Disagree to 4 = Strongly Agree), with higher scores indicating higher teacher job satisfaction. The Cronbach’s alpha coefficient for the total scale is 0.839, while the alpha coefficients for the “Job satisfaction with work environment” and “Job satisfaction with profession” dimensions are 0.795 and 0.761, respectively. The confirmatory factor analysis (CFA) of job satisfaction indicated good model fit (RMSEA = 0.085, CFI = 0.951, TLI = 0.909, SRMR = 0.035). The standardized factor loadings ranged from 0.664 to 0.812. This suggests that the scale adequately reflects teachers’ job satisfaction.

#### 3.2.3 Teacher-student relationship

The teacher-student relationship comprises four items, such as “Teachers and students usually get along well with each other” and “Most teachers believe that students’ well-being is important.” Several previously published papers have also operationalized teachers’ perceptions of the teacher-student relationship in TALIS in this manner (e.g., Lopes and Oliveira, 2020; Wang, 2025). All items use a four-point Likert scale, with options ranging from “1 = Strongly Disagree” to “4 = Strongly Agree”; higher scores indicate a better teacher-student relationship. In this study, the Cronbach’s alpha coefficient for this scale was 0.929. Confirmatory factor analysis of the Teacher-Student Relationship scale indicated good model fit (RMSEA = 0.047, CFI = 0.992, TLI = 0.976, SRMR = 0.011). The standardized factor loadings ranged from 0.852 to 0.888. This suggests that the scale adequately reflects the Teacher-Student Relationship.

#### 3.2.4 Teacher self-efficacy

This study utilized the Teacher Self-Efficacy Scale from the TALIS 2024 questionnaire. This scale comprises three subscales: Efficacy in Classroom Management, Efficacy in Instruction, and Efficacy for Student Engagement. The Efficacy in Instruction dimension includes four items, such as “Use a variety of assessment strategies.” Similarly, the Efficacy in Classroom Management dimension includes four items, such as “Control disruptive behavior in the classroom.” Additionally, the Efficacy for Student Engagement dimension includes four items, such as “Help my students value learning.” All items are scored using a four-point Likert scale ranging from “1 = Not at all” to “4 = A lot,” with higher scores indicating stronger teacher self-efficacy. In this study, the Cronbach’s alpha coefficients for the total scale were 0.838. For the subscales Efficacy in Classroom Management, Efficacy in Instruction, and Efficacy for Student Engagement, the Cronbach’s alpha coefficients were 0.883, 0.864, and 0.864, respectively. Confirmatory Factor Analysis indicated good model fit (RMSEA = 0.076, CFI = 0.937, TLI = 0.913, SRMR = 0.045), with standardized factor loadings ranging from 0.688 to 0.833. This suggests that the scale adequately reflects teacher self-efficacy.

To control for the influence of potential confounding factors, and in reference to existing literature (Klassen and Chiu, 2010; Matsuoka, 2015; Wang et al., 2020), variables such as teacher gender, teaching experience, and school location (urban or rural) were included as covariates in the main analysis. By incorporating these covariates into the statistical model, the study can present more reliable results.

### 3.3 Data analysis

All data in this study were analyzed using Stata 17.0 and Mplus 8.3. First, a common method bias test was conducted using Stata 17.0. Subsequently, descriptive statistics and correlation analyses were performed. Finally, the researchers used Mplus 8.3 to conduct confirmatory factor analysis to assess the reliability and validity of the scales, and to construct a structural equation model to evaluate the relationships among growth mindset, job satisfaction, teacher-student relationship, and self-efficacy. Additionally, the study employed the Bootstrap method to examine the mediating roles of teacher-student relationship and self-efficacy in the relationship between teachers’ growth mindset and job satisfaction. When using Mplus for parameter estimation and standard error calculation, the study used the teacher weights (TCHWGT) and school identifiers (IDSCHOOL) from the TALIS dataset as weighting and clustering variables to address unequal sampling probabilities and non-independence among teachers (Özdemir et al., 2023).

## 4 Results

### 4.1 Common method bias test

Since the data in this study were collected through teacher self-reports, common method bias may be a concern. To minimize this risk, the OECD implemented several procedural safeguards when designing and administering the 2024 TALIS survey, including ensuring respondent anonymity, using varied item wording, and employing different scale anchors. At the statistical level, first, this study employed Harman’s single-factor test to assess common method bias (Harman, 1976). The results indicated that six factors with eigenvalues greater than 1 were extracted. The first factor explained only 31.41% of the variance, which is below the 40% critical threshold (Podsakoff and Organ, 1986), suggesting that no significant common method bias exists in this study. Second, the unmeasured latent method construct (ULMC) technique was used to re-examine common method bias. A comparison of model fit indices before and after incorporating the method factor showed that: *∆*CFI = 0.022, ∆TLI = 0.022, ∆RMSEA = 0.013, ∆SRMR = 0.003. The model fit did not improve significantly. Therefore, it can be concluded that there is no serious common method bias in this study.

### 4.2 Measurement model assessment

As shown in Table 1, the reliability and validity of all constructs meet good standards: the composite reliability (CR) of each construct ranges from 0.853 to 0.946, all exceeding the recommended threshold of 0.70 (Cheung et al., 2024), indicating that the scale possesses ideal internal consistency reliability. Regarding convergent validity, the average variance extracted (AVE) for each construct ranged from 0.549 to 0.766, all meeting or exceeding the critical threshold of 0.50 (Cheung et al., 2024), indicating that the latent variables explain a good proportion of the variance in their observed indicators. Regarding discriminant validity, the Heterogeneity-to-Homogeneity Ratio (HTMT) between all pairs of constructs ranged from 0.211 to 0.766, all falling below the strict threshold of 0.85 (Fornell and Larcker, 1981), confirming satisfactory discriminant validity among the constructs. In summary, the measurement model in this study possesses sound psychometric properties and is suitable for subsequent structural equation modeling analysis.

**Table 1:** Results of the measurement model assessment.

Variables	CR	AVE	HTMT			
			1	2	3	4
1. Growth mindset	0.853	0.661	1			
2. Teacher-student relationship	0.929	0.766	0.259	1		
3. Teacher self-efficacy	0.946	0.592	0.211	0.664	1	
4. Job satisfaction	0.879	0.549	0.402	0.524	0.366	1

### 4.3 Descriptive statistics and correlations

Table 2 presents the means, standard deviations, and correlation coefficients for the four main variables in this study. A growth mindset was found to be significantly positively correlated with teacher job satisfaction (*r* = 0.336, *p* < 0.001), teacher-student relationship (*r* = 0.228, *p* < 0.001), and self-efficacy (*r* = 0.203, *p* < 0.001). The teacher-student relationship was significantly positively correlated with teacher job satisfaction (*r* = 0.432, *p* < 0.001) and self-efficacy (*r* = 0.463, *p* < 0.001). Furthermore, a significant positive correlation was found between self-efficacy and teacher job satisfaction (*r* = 0.332, *p* < 0.001). These findings preliminarily support the research hypotheses.

**Table 2:** Descriptive statistics and correlation analysis.

Variables	M	S D	1	2	3	4
1. Growth mindset	2.481	0.647	1			
2. Teacher-student relationship	3.469	0.477	0.228^***^	1		
3. Teacher self-efficacy	3.489	0.489	0.203^***^	0.463^***^	1	
4. Job satisfaction	3.003	0.445	0.336^***^	0.432^***^	0.332^***^	1

***p < 0.001; M, mean; SD, standard deviation.

### 4.4 Hypothesis testing

Based on the research hypotheses, this study constructed a structural equation model in which a growth mindset serves as the independent variable, teacher job satisfaction as the dependent variable, and teacher-student relationships and self-efficacy as mediating variables (a chained mediation model). This model aims to explore the mechanism through which a growth mindset influences teacher job satisfaction. To enhance model reliability and parsimony, job satisfaction and teacher self-efficacy were parceled using the internal-consistency approach. For each construct, the items within a subdimension were averaged to form a single parcel, and this mean score served as the observed indicator of the latent factor. In addition to this hypothesized model, a competing model with parallel mediation was constructed (i.e., removing the path from teacher-student relationship to self-efficacy, allowing both to influence job satisfaction in parallel) to test the superiority of the hypothesized model. A comparison of model fit revealed that the difference in chi-square values (Δχ^2^ = 217.714; Δdf = 1) was statistically significant (*p* < 0.001), indicating that the hypothesized model is significantly superior to the competing model. Furthermore, the fit indices of the hypothesized model (CFI = 0.984, TLI = 0.980, RMSEA = 0.033, SRMR = 0.030) were all superior to those of the competing model (CFI = 0.953, TLI = 0.942, RMSEA = 0.057, SRMR = 0.109). Based on the results of the above comparison tests, the observed data fit best with the proposed model; therefore, the chained mediation model is accepted.

**Figure 2:**
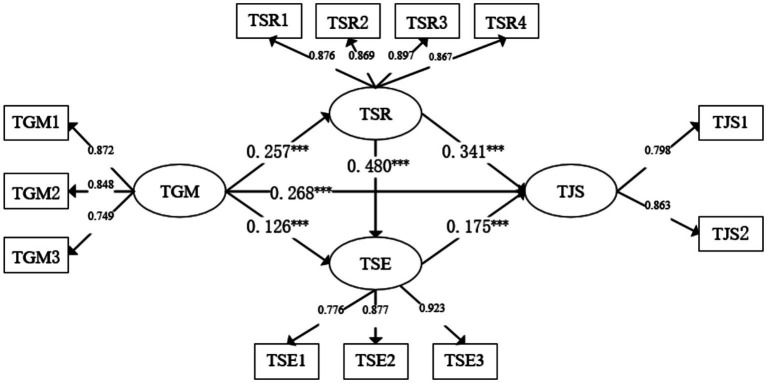
SEM of growth mindset and teacher job satisfaction. TGM, teacher’s growth mindset; TSR, teacher-student relationship; TSE, teacher self-efficacy; TJS, teacher job satisfaction. Standardized coefficients are reported. ^***^p < 0.001.

Figure 2 shows that teachers’ growth mindset is significantly and positively correlated with job satisfaction, teacher-student relationship, and self-efficacy (*β* = 0.268, *p* < 0.001; *β* = 0.257, *p* < 0.001; *β* = 0.*126*, *p* < 0.001). Teacher-student relationship was also significantly positively correlated with teacher job satisfaction and self-efficacy (*β* = 0.341, *p* < 0.001; *β* = 0.480, *p* < 0.001). There was a significant positive correlation between teacher self-efficacy and job satisfaction (β = 0.175, *p* < 0.001). Therefore, Hypothesis H1 is supported.

The study continued to employ the bias-corrected percentile Bootstrap method, with 5,000 sample repetitions, to examine the mediating effects of teacher-student relationship and self-efficacy on the relationship between growth mindset and teacher job satisfaction. As shown in Table 3, the direct effect of teachers’ growth mindset on job satisfaction was 0.268, with a 95% confidence interval (CI) of [0.199, 0.338]. Since the 95% CI does not include 0, this indicates a significant direct effect of teachers’ growth mindset on job satisfaction. The effect size of the teacher-student relationship on the relationship between growth mindset and teacher job satisfaction is 0.088, with a 95% confidence interval of [0.062, 0.113] that excludes 0, indicating that the teacher-student relationship partially mediates this relationship. Similarly, the effect size of self-efficacy between growth mindset and teacher job satisfaction is 0.022, with a 95% confidence interval of [0.009, 0.035] that does not include 0, indicating that self-efficacy partially mediates the relationship between the two. Furthermore, the effect size of the teacher-student relationship and self-efficacy on the relationship between growth mindset and teacher job satisfaction is 0.022, with a 95% CI of [0.012, 0.032] that excludes 0, indicating that the teacher-student relationship and self-efficacy play a chained mediating role between the two variables. Therefore, Hypotheses H4, H7, and H9 are supported.

**Table 3:** Test of mediating effect.

Path	Effect	SE	95% CI		Ratio
			Lower	Upper	
Total effect	0.399	0.034	0.333	0.466	
Direct effect	0.268	0.035	0.199	0.338	67.17%
Indirect effect	0.131	0.015	0.101	0.161	32.83%
TGM → TSR → TJS	0.088	0.013	0.062	0.113	22.06%
TGM → TSE → TJS	0.022	0.007	0.009	0.035	5.51%
TGM → TSR → TSE → TJS	0.022	0.005	0.012	0.032	5.51%

TGM, teacher’s growth mindset; TSR, teacher-student relationship; TSE, teacher self-efficacy; TJS, teacher job satisfaction. There is a slight discrepancy between the sum of the three specific indirect effects. Moreover, the total indirect effect is due to rounding.

## 5 Discussion

Based on the TALIS 2024 data from Shanghai, China, this study examines the complex relationships among teachers’ growth mindset, teacher-student relationship, self-efficacy, and job satisfaction. The results not only confirm a significant positive correlation between growth mindset and teacher job satisfaction but also reveal multiple mediating roles for teacher-student relationship and teacher self-efficacy, particularly a chained mediation pathway between these two factors. These findings provide a more nuanced explanation for understanding the intrinsic link between teachers’ growth mindset and job satisfaction. However, due to the cross-sectional nature of the data, all findings and interpretations presented below are based on associations and correlational patterns. no causal direction can be inferred among the variables.

First, the growth mindset of teachers in Shanghai, China, is at a moderate level. This result is highly consistent with a recent meta-analysis focusing on Chinese samples. Sun and Wang (2025) synthesized data from 42 independent samples encompassing more than 50,000 Chinese participants and found that, although Chinese individuals overall tend to endorse a growth mindset, the effect size is only small to moderate, significantly lower than that observed in Western samples such as those from the United States. Existing research, including our own, indicates that in the Chinese cultural context, beliefs about effort and beliefs about the malleability of intelligence are relatively independent: individuals may firmly hold that effort is a virtue, yet this does not necessarily mean they believe that intelligence is inherently changeable (Wang and Sun, 2025). At the same time, given the positive impact of a growth mindset on teachers, this moderate level also indicates that there is still room for improvement in fostering a growth mindset among Chinese teachers, which provides a practical foundation for implementing targeted interventions to promote a growth mindset in the future.

Second, the positive correlation between growth mindset and teacher job satisfaction aligns with Dweck’s (2006) theory. This theory suggests that a growth mindset is the belief that abilities can be developed through dedication and effort. This mindset fosters resilience and enthusiasm for work. These qualities, in turn, may be associated with teacher job satisfaction. Previous research indicates that a growth mindset may influence how individuals cope with interpersonal conflicts (Chan et al., 2014), fostering trust and a healthy work environment, thereby improving interpersonal relationships and enhancing job satisfaction. Furthermore, when faced with setbacks and adversity, individuals with a growth mindset tend to adopt a mastery-oriented coping style, characterized by persistent effort, the search for problem-solving strategies, and a commitment to completing challenging tasks (Dweck and Yeager, 2019). Regarding the affective outcomes of job satisfaction, evidence suggests that mastery-oriented individuals may derive greater satisfaction and enjoyment from the effort required to achieve their goals (Janssen and Van Yperen, 2004). Consequently, teachers with a growth mindset may experience higher levels of job satisfaction.

Third, the teacher-student relationship exerts a significant mediating effect between growth mindset and job satisfaction. The significant mediating effect of the teacher-student relationship underscores the importance of relatedness need fulfillment, as articulated in self-determination theory (Deci et al., 2017). In Chinese educational tradition, the teacher-student relationship is deeply embedded in Confucian heritage, which positions teachers not merely as knowledge transmitters but as moral exemplars and life-long mentors (shi sheng ru fu), blending professional authority with parental-like care and mutual obligation (Ho, 1998; Jin and Cortazzi, 2006). This culturally scripted bond makes the quality of teacher-student relationship a particularly potent source of teachers’ professional fulfillment. Consistent with this, research on Chinese teacher samples has identified a close, caring teacher-student relationship as a robust correlate of teacher job satisfaction and a buffer against turnover intentions (Liu et al., 2023). When teachers experience an emotional connection with students, manifested as respect, trust, positive responsiveness, and a warm atmosphere in daily interactions, both their intrinsic motivation and willingness to engage professionally are strengthened (Heinla and Kuurme, 2024). However, not all teachers are equally effective at building high-quality teacher-student relationship. In this process, a growth mindset emerges as a critical individual difference variable. In the high-pressure educational landscape of Shanghai, where academic competition can intensify interpersonal tensions between teachers and students, teachers with a high growth mindset are more likely to view friction in teacher-student interactions or student resistance as communication issues that can be improved, rather than as evidence of unchangeable inherent student traits. This belief prompts them to adjust their interaction styles proactively and to attempt to understand students’ emotional and behavioral motivations from students’ perspectives, thereby increasing the likelihood of establishing psychological bonds based on mutual respect and trust (Dweck and Leggett, 1988). Conversely, teachers with a fixed mindset tend to attribute relationship tensions to students’ problematic traits or external environmental factors, leading them to adopt avoidance, blame, or passive-aggressive strategies. This results in a vicious cycle of conflict or alienation in teacher-student relationship (Dweck and Leggett, 1988). Therefore, a growth mindset enhances the quality of teacher-student relationship by guiding teachers to address interpersonal challenges constructively, while supportive, warm teacher-student relationship, as critical psychological resources, directly improves teachers’ job satisfaction.

Fourth, this study further confirms that self-efficacy plays a significant mediating role between teachers’ growth mindset and job satisfaction. According to self-determination theory, the core reason a growth mindset enhances teacher self-efficacy is that it satisfies teachers’ basic psychological needs, particularly the need for competence (Bandura, 1978). Specifically, teachers with a growth mindset view teaching setbacks as opportunities for improvement rather than definitive judgments of their ability. This belief makes it easier for them to experience a sense of competence by achieving desired outcomes through effort in their teaching practice. Each instance of effectively overcoming difficulties directly reinforces the perception of their ability to teach well, thereby enhancing self-efficacy. Once self-efficacy is strengthened, it further promotes job satisfaction. According to Self-Determination Theory, the fulfillment of a sense of competence is a core prerequisite for intrinsic motivation and psychological well-being. Teachers with high self-efficacy experience a stronger sense of competence in their work, and this sense of competence is intertwined with autonomy and a sense of belonging (Bandura, 1978). When teachers are confident in their ability to handle challenges, they are more inclined to internalize teaching behaviors, independently choose teaching strategies, and seek peer feedback and collaboration, thereby fulfilling their needs for autonomy and belonging. The simultaneous fulfillment of these three fundamental psychological needs is associated with a systematic enhancement of teachers’ job satisfaction (Deci et al., 2017).

Fifth, the teacher-student relationship and self-efficacy constitute a chain of mediating effects linking a growth mindset to job satisfaction. This finding integrates variables from three levels, namely individual beliefs, interpersonal environment, and self-perception, into a chain, deepening our understanding of the mechanisms underlying the growth mindset. According to Bandura’s social cognitive theory, the development of self-efficacy primarily stems from four sources of information: direct experience, vicarious experience, verbal persuasion, and emotional arousal (Bandura, 1978). In teacher-student interactions, teachers may perceive positive feedback from students. This feedback can include increased classroom participation, improved academic performance, or emotional trust and reliance. These direct experiences convey the message that their teaching is effective and they can influence students. As a result, their belief in their own teaching abilities is reinforced. At the same time, students’ respect and verbal encouragement constitute a form of verbal persuasion, while the positive emotional atmosphere fostered by a warm, harmonious teacher-student relationship further enhances teachers’ professional confidence through emotional arousal mechanisms (Wang, 2025). In other words, the teacher-student relationship is not only an interpersonal resource that satisfies relational needs but also a core channel through which teachers obtain information confirming their competence. When a growth mindset guides teachers to build supportive teacher-student relationship proactively, they are better able to gather evidence of their teaching efficacy from daily interactions, thereby enhancing their self-efficacy (Xu and Qi, 2019). Extensive research has confirmed the significant role of self-efficacy. Self-efficacy refers to an individual’s confidence in their ability to accomplish specific tasks. It directly drives teachers’ work engagement, pedagogical innovation, and career persistence. It also serves as a strong predictor of job satisfaction (Perera et al., 2018). Therefore, there is a positive correlation between a growth mindset and job satisfaction, and this correlation may be sequentially mediated by teacher-student relationship and teacher self-efficacy. However, it is worth noting that all interpretations above are constrained by the cross-sectional design. The observed chain mediating effects are statistically consistent with the proposed model, but alternative directional or reciprocal relationships cannot be ruled out.

Sixth, it should be noted that when interpreting these indirect effects, their effect sizes must be critically evaluated. Although the chain mediation of teacher-student relationship and self-efficacy and the independent mediation of self-efficacy both achieved statistical significance, the standardized effect size for each was only 0.022, accounting for a mere 5.51% of the total effect, respectively. In other words, while the self-efficacy pathway and the chain pathway involving self-efficacy provide empirical support for the competence need satisfaction pathway in self-determination theory, their actual incremental explanatory power is quite limited and should not be characterized as a core mechanism influencing teacher job satisfaction. This finding reminds us that when translating research into practical recommendations, we should not overstate the applied value of small indirect effects, but rather guide schools and policymakers to prioritize limited resources on interventions that can directly improve teachers’ growth mindset and teacher-student relationship.

Finally, notably, although Self-Determination Theory (SDT) posits three basic psychological needs—autonomy, competence, and relatedness—the present study only examined relatedness (teacher-student relationship) and competence (self-efficacy) while autonomy was not included in the empirical model. This omission was primarily driven by data constraints: the TALIS 2024 Shanghai dataset does not contain a scale that directly measures teachers’ psychological need for autonomy as conceptualized within SDT. The available items concerning teacher autonomy in TALIS predominantly refer to decision-making latitude over curriculum, assessment, and school policies, which capture job autonomy or structural empowerment rather than the experiential quality of volition and self-endorsement that defines the need for autonomy in SDT. Consequently, it was not feasible to incorporate autonomy as a parallel mediator in the current structural equation model. Readers should therefore bear in mind that our model tests only two of the three SDT needs, and the complete theoretical picture awaits future research that includes validated measures of autonomy need satisfaction. Such work would allow a full examination of whether and how the satisfaction of all three basic needs jointly transmits the association between growth mindset and teacher job satisfaction.

## 6 Theoretical and practical implications

Based on Implicit Theories and Self-Determination Theory, this study systematically reveals the underlying mechanisms through which a growth mindset influences teacher job satisfaction via multiple mediating pathways involving teacher-student relationship and self-efficacy. First, it validated the independent mediating role of teacher-student relationship, elucidating how a growth mindset enhances job satisfaction by guiding teachers to construct interpersonal interactions and actively satisfy their relational needs. Second, the study confirmed the independent mediating role of self-efficacy, demonstrating that a growth mindset is positively related to higher self-efficacy through the fulfillment of teachers’ need for competence, and self-efficacy is, in turn, positively associated with job satisfaction. Most importantly, the study integrated the chained mediating effects of teacher-student relationships and self-efficacy. It incorporated three dimensions into a unified analytical framework. These dimensions are individual beliefs, interpersonal environment, and self-perception. In this framework, a growth mindset first drives the formation of supportive teacher-student relationships. High-quality teacher-student relationships then strengthen teacher self-efficacy by providing sources of efficacy information, such as direct experience and emotional arousal. This process ultimately translates into higher levels of job satisfaction. This finding offers a more nuanced theoretical explanation for understanding the complex pathways through which a growth mindset influences teachers’ job well-being.

The findings of this study offer clear practical guidance for enhancing teachers’ job satisfaction. First, schools should focus on cultivating teachers’ growth mindsets through specialized workshops, case studies, and growth-oriented evaluation systems, helping teachers view teaching setbacks as opportunities for skill development, thereby directly enhancing their professional well-being. Second, given the mediating role of teacher-student relationship, administrators should provide teachers with training in specific communication strategies, such as active listening and nonviolent communication, encouraging them to view interpersonal friction through a growth mindset and proactively adjust their interaction styles to establish psychological bonds of mutual respect and trust. Third, addressing the mediating role of self-efficacy, schools can systematically enhance teachers’ sense of competence through targeted feedback, moderately challenging tasks (e.g., cross-grade teaching or project-based learning designs), and peer observation. Finally, based on the finding that the chain mediation effect exists but has a small effect size, we recommend that schools and policymakers prioritize resource allocation accordingly: they should first focus on directly cultivating teachers’ growth mindset and positive teacher-student relationship, while treating the enhancement of teacher self-efficacy as a supplementary, consolidating intervention target. Comprehensive interventions could follow a sequential strategy of first improving core beliefs and the interpersonal environment, and then using positive feedback to reinforce self-efficacy; however, self-efficacy should not be regarded as a driver of equal importance to growth mindset, in order to ensure intervention effectiveness and resource efficiency.

## 7 Limitations and future directions

The study has several limitations that require further refinement in future research. First, the sample primarily consisted of teachers from the educationally advanced region of Shanghai; therefore, the results may not be directly generalizable to teachers in less developed regions or those with different educational contexts. Future studies could adopt multi-center, cross-regional sampling designs to test the stability of relationships among variables across different geographical and cultural contexts. Second, this study employed a cross-sectional survey design, which does not permit strict causal inference regarding the relationships among growth mindset, self-efficacy, and job satisfaction. Future research could utilize longitudinal tracking or experimental intervention designs further to clarify the directionality and dynamic mechanisms of these relationships. Furthermore, all research data were derived from teacher self-reports. While these reflect subjective psychological experiences, they may be influenced by factors such as social desirability and common method bias, thereby compromising the objectivity and accuracy of the results. Future research could attempt to incorporate multi-source, multi-method assessment tools, such as peer evaluations, classroom behavior observations, or performance records, to enhance the reliability and validity of the data and the robustness of the research conclusions.

## 8 Conclusion

Based on Self-Determination Theory and using TALIS 2024 data from Shanghai teachers, this study reveals that a growth mindset not only directly and positively predicts teacher job satisfaction but also exerts indirect effects through the independent mediating roles of teacher-student relationship and teacher self-efficacy, as well as through a chain mediation path of “teacher-student relationship → self-efficacy.” The more teachers believe that abilities are malleable, the more readily they build supportive teacher-student relationships, from which they derive efficacy information and strengthen their sense of teaching competence, ultimately enhancing their satisfaction with both the profession and the work environment. By integrating individual beliefs, interpersonal environment, and self-perception into a unified analytical framework, this chain mechanism refines the psychological pathways through which a growth mindset enhances teachers’ professional well-being and offers important insights for understanding teacher retention in high-pressure educational contexts. Practically, priority should be given to cultivating a growth mindset and improving teacher-student relationships, with self-efficacy enhancement serving as a consolidating strategy, thereby creating a virtuous cycle that sustainably promotes teacher job satisfaction.

## Data Availability

Publicly available datasets were analyzed in this study. This data can be found: https://www.oecd.org/en/data/datasets/talis-2024-database.html.
